# Supplementary figures and images for: Human milk cortisol and immune factors over the first three postnatal months: Relations to maternal psychosocial distress
Source: PLoS One. 2020 May 21;15(5):e0233554. doi: 10.1371/journal.pone.0233554 (PMC7241837; doi:10.1371/journal.pone.0233554)

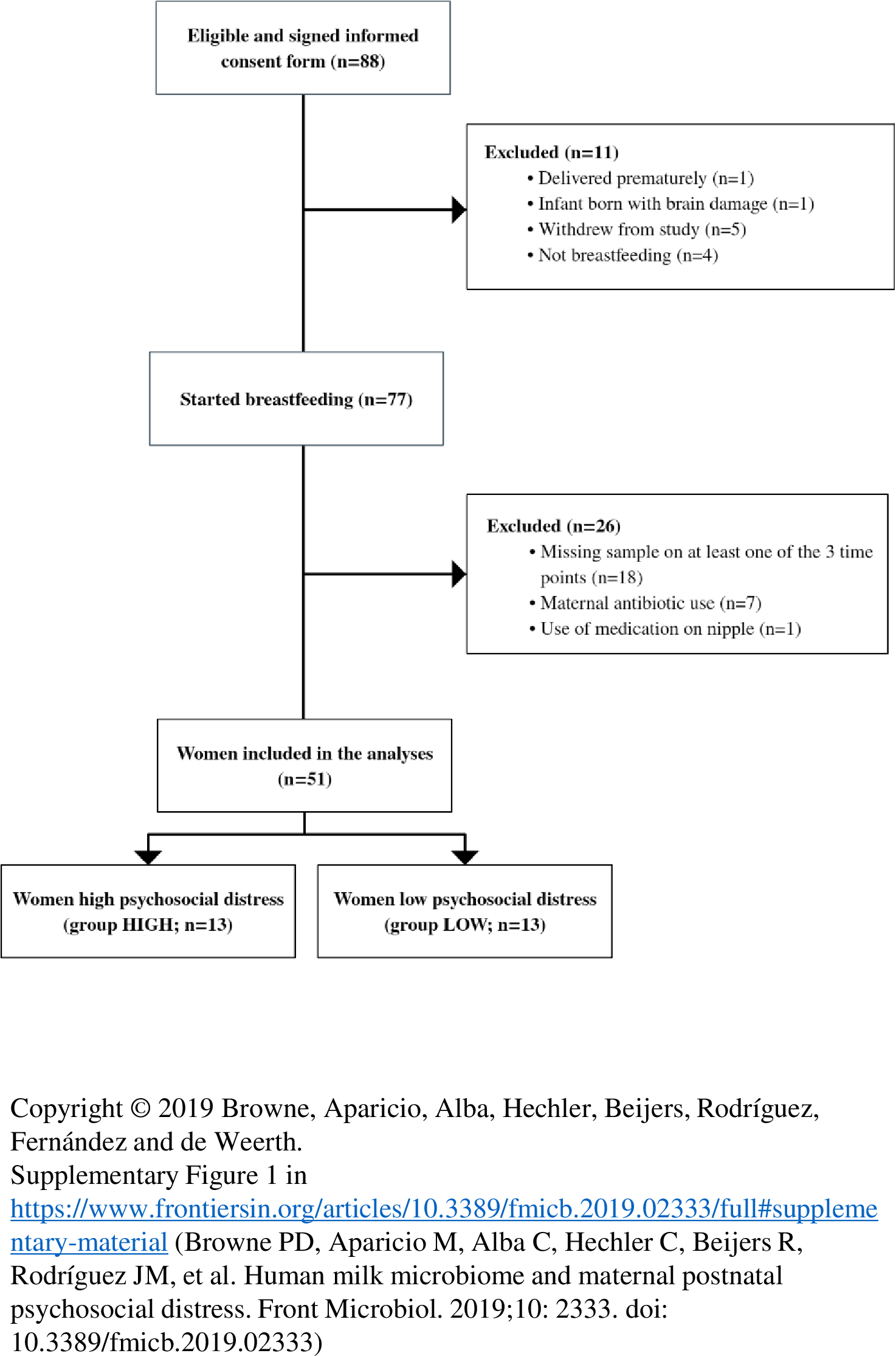

Supplement: S1 Fig — (TIF) [file pone.0233554.s001.tif]

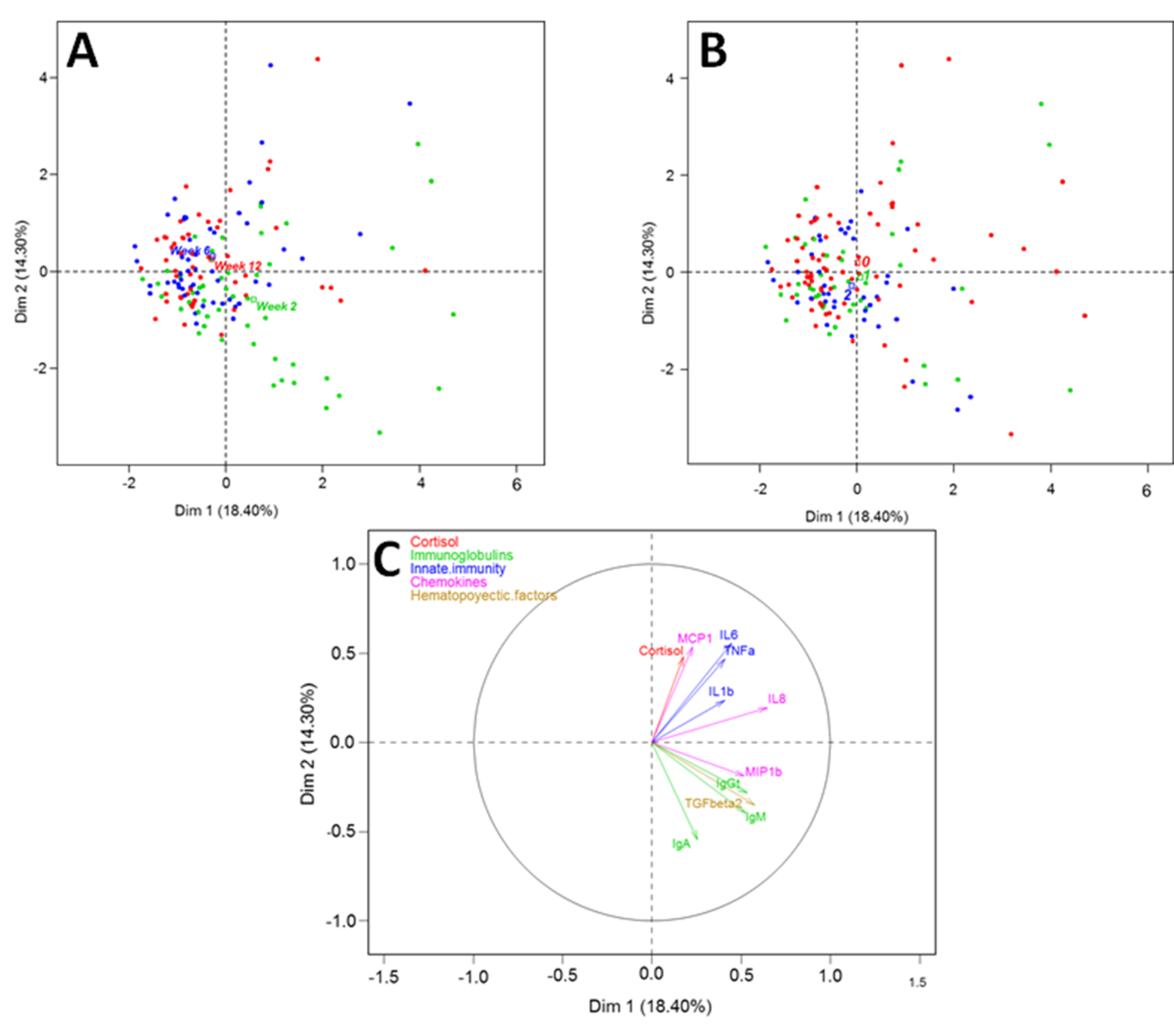

Supplement: S2 Fig — One sample was excluded because it determined the strong aggregation of the rest of the samples. Individual factor maps for milk samples, where each point represents a milk sample: (A) samples taken at week 2 postpartum are colored in green, at week 6 postpartum are colored in blue, and at week 12 postpartum are colored in red; (B) samples from women with low (0, red), medium (1, green) and high (2, blue) maternal postnatal psychosocial distress. (C) Correlation circle showing the active (cos2>0.2) variables (arrows) contributing to the spatial distribution of the milk samples represented in the upper graph (A). Arrows pointing in the same direction are positively correlated, in the opposite direction are negatively correlated, and unrelated when they are orthogonal (90º). (TIF) [file pone.0233554.s002.tif]

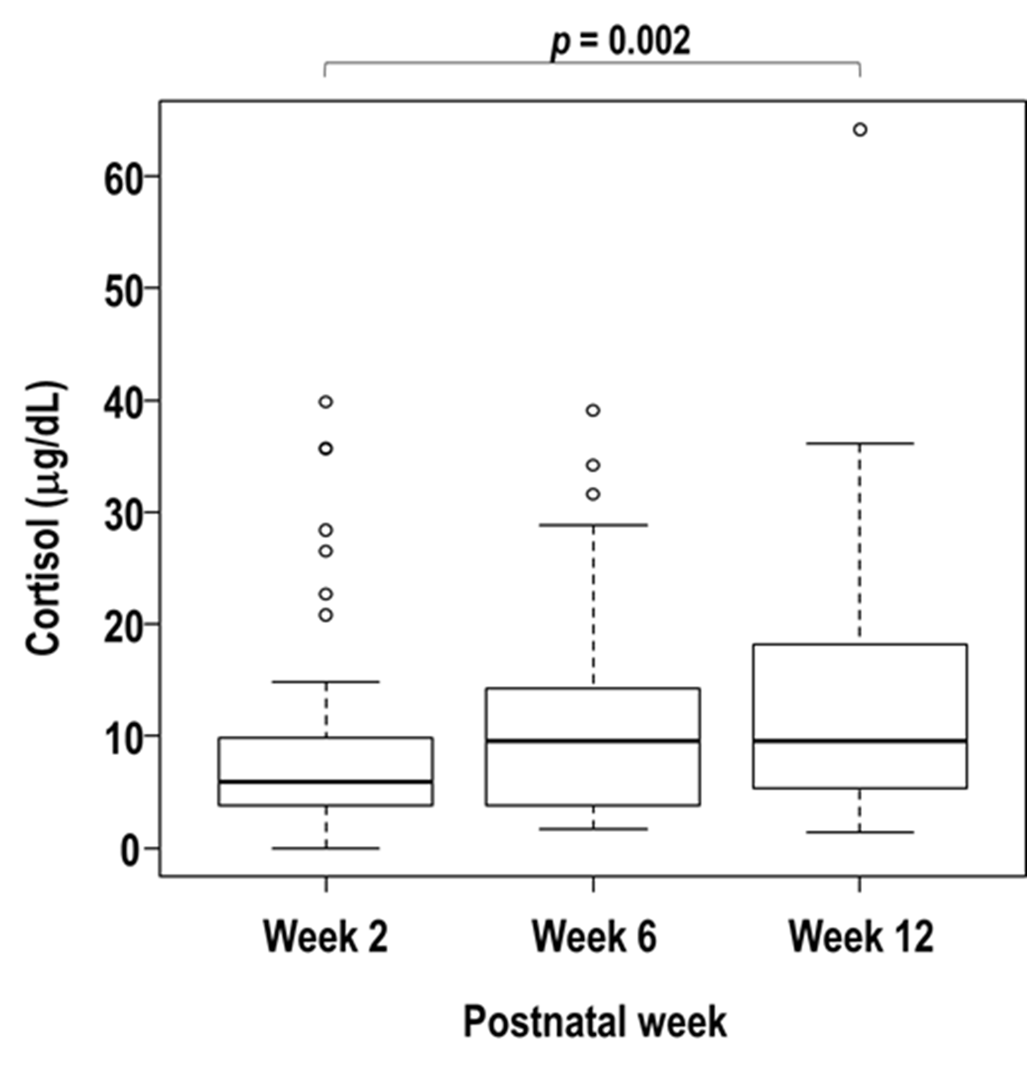

Supplement: S3 Fig — White circles represent outliers (>1.5× IQR). The bar on the top indicates significant differences between the cortisol concentration in milk samples taken at weeks 2 and 12 (Friedman’s non parametric repeated measures comparison followed by post-hoc Nemenyi tests). (TIF) [file pone.0233554.s003.tif]
