# Supplementary material for: Human milk cortisol and immune factors over the first three postnatal months: Relations to maternal psychosocial distress
Source: PLoS One. 2020 May 21;15(5):e0233554. doi: 10.1371/journal.pone.0233554 (PMC7241837; doi:10.1371/journal.pone.0233554)
Supplement: S1 Table — (DOCX) [file pone.0233554.s004.docx]

| **Immunological factor** | **Week 2** | **Week 6** | **Week 12** | **p-valueǂ** |
| --- | --- | --- | --- | --- |
| **Group LOW (n = 13)** | | | | |
| ***Immunoglobulins* (mg/L)** | | | | |
| IgA (×10^3^) | 1.81 (1.26-2.48)^a^ | 1.45 (1.07-1.79)^b^ | 1.20 (1.14-1.82)^b^ | 0.002 |
| IgGt | 47.70 (34.60-85.91) | 43.00 (30.36-58.44) | 48.23 (37.31-51.69) | 0.368 |
| IgM | 60.64 (54.70-93.13)^a^ | 29.12 (18.35-55.30)^b^ | 33.06 (25.38-39.93)^b^ | <0.001 |
| ***Innate immunity* (ng/L)** | | | | |
| IL1β | 0.68 (0.27-2.43) | 0.63 (0.29-0.79) | 0.20 (0.11-0.31) | 0.112 |
| IL6 | 10.04 (7.51-14.88) | 4.59 (2.63-22.05) | 1.53 (1.19-2.09) | - |
| TNFa | 2.94 (1.01-4.95) | 1.97 (0.86-3.04) | 2.86 (0.74-5.02) | 0.135 |
| ***Chemokines* (ng/L)** | | | | |
| IL8 | 25.81 (11.04-82.97)^a^ | 6.91 (4.50-27.62)^b^ | 10.24 (9.27-17.05)^ab^ | 0.012 |
| MCP1 | 267.63(133.08-621.50)^a^ | 118.12(90.21-218.63)^b^ | 44.45 (35.59-53.15)^b^ | 0.039 |
| MIP1β | 34.21 (7.74-68.07)^a^ | 10.36 (4.56- 15.67)^b^ | 3.63 (1.84-4.01)^b^ | 0.012 |
| GROα (×10^3^) | 3.8 (1.11-4.07) | 0.73 (0.17-6.38) | 0.77 (0.46-2.06) | 0.926 |
| ***Hematopoyetic* *factors* (μg/L)** | | | | |
| EGF | 4.50 (3.60-5.23)^a^ | 4.04 (3.56-4.92)^b^ | 3.78 (2.67-5.35)^ab^ | 0.023 |
| TGFβ_2_ | 2.17 (1.65-2.72) | 1.21 (0.84-2.61) | 2.16 (0.96-2.57) | 0.125 |
| **Group HIGH (n = 13)** | | | | |
| ***Immunoglobulins* (mg/L)** | | | | |
| IgA (×10^3^) | 1.91 (1.06-2.08) | 1.51 (0.96-2.01) | 1.56 (1.10-1.68) | 0.368 |
| IgGt | 52.11 (44.00-67.20)^a^ | 36.35 (32.58-60.73)^ab^ | 42.00 (30.72-52.72)^b^ | 0.058 |
| IgM | 78.72(58.67-100.93)^a^ | 45.29(39.79-79.09)^b^ | 42.90(23.50-74.44)^b^ | 0.002 |
| ***Innate immunity* (ng/L)** | | | | |
| IL1β | 0.52 (0.33-1.42) | 0.67 (0.60-0.95) | 0.35 (0.21-2.38) | 0.067 |
| IL6 | 7.84 (5.87-46.34) | 4.49 (3.62-7.50) | 2.70 (1.36-5.27) | 0.135 |
| TNFα | 1.95 (1.09-4.95) | 3.01 (1.58-4.00) | 2.84 (1.38-3.33) | 0.748 |
| ***Chemokines* (ng/L)** | | | | |
| IL8 | 13.27 (5.77-53.81) | 8.65 (6.21-48.12) | 14.46 (7.42-34.67) | 0.116 |
| MCP1 | 372.17(148.60-515.08)^a^ | 110.14(40.64-123.47)^b^ | 43.08(19.53-60.69)^b^ | 0.039 |
| MIP1β | 14.71 (2.59-39.98)^a^ | 5.29 (1.55-14.68)^b^ | 2.63 (1.18-14.57)^b^ | 0.005 |
| GROα (×10^3^) | 4.12 (0.37-5.93)^a^ | 0.25 (0.18-0.99)^b^ | 0.44 (0.17-0.92)^b^ | <0.001 |
| ***Hematopoyetic* *factors* (μg/L)** | | | | |
| EGF | 5.75 (5.50-6.88) | 5.28 (4.12-6.24) | 4.64 (4.00-5.54) | 0.058 |
| GFβ_2_ | 2.35 (1.32-3.68) | 1.05 (0.72-1.45) | 2.22 (1.71-2.74) | 0.058 |

Concentration of immune factors was expressed as medians and IQR.

**^ǂ^** Friedman rank sum tests were used to evaluate differences in the concentration of each immune factor in each group across time.
