# Supplementary material for: Human milk cortisol and immune factors over the first three postnatal months: Relations to maternal psychosocial distress
Source: PLoS One. 2020 May 21;15(5):e0233554. doi: 10.1371/journal.pone.0233554 (PMC7241837; doi:10.1371/journal.pone.0233554)
Supplement: Attachment — Submitted filename: Response to Reviewers_31037R1.docx [file pone.0233554.s007.docx]

Madrid, 7 May 2020

Dear editors of PLOS ONE,

We are resubmitting our revised research manuscript PONE-D-19-31037R2 “Human milk

cortisol and immune factors over the first three postnatal months: relations to maternal

psychosocial distress” by Marina Aparicio, Pamela Browne, Christine Hechler, Roseriet

Beijers, Juan Miguel Rodríguez, Carolina de Weerth and Leonides Fernandez for publication

in PLOS ONE.

We are glad the reviewers found our revised manuscript (almost) ready for publication.

Below we answer Reviewer 2’s remaining two points.

Sincerely,

Carolina de Weerth and Leonides Fernández, also on behalf of the remaining authors

Reviewer #2: The authors have sufficiently acknowledged most of the comments.
I still have two suggestions to complete the revision:

1. Absence of evidence is not evidence of absence. Considering all the limitations related to the sample size and the actual design, I would like the conclusions to be phrased differentially. Instead of stating that "Immune factor concentrations in milk do not seem influenced by natural variations in maternal distress" I would like to see a comment statin that "We found no evidence on ... " or "Our data did not support ...." There are so many limitations and restrictions underlying this paper, I would like the conclusion to comprise more directly the many uncertainties related to the absence of the expected association.

*We agree with the reviewer and replaced our concluding sentence in the Abstract by: “In the current study we found no evidence for an association between natural variations in maternal distress and immune factor concentrations in milk.” (lines 51-52)*

*We additionally adapted the sentence starting on line 607 of the Discussion as follows: “The current study added to this emerging literature by showing that immune factor concentrations are not always associated to natural variations in maternal distress, at least in healthy populations such as the current one.” This sentence replaces the sentence: “The current study added to this emerging literature by showing that immune factor concentrations in milk do not seem influenced by natural variations in maternal distress, at least in healthy populations such as the current one.”*

2. The implications of my point #5 should also be discussed in the discussion section in addition to the revised results section. the fact that the important variation in sampling time was biased by the maternal condition of psychological symptoms should be discussed clearly.

*We now adapted the sentence starting on line 590 to “Women with higher psychosocial distress reported later waking times, as well as longer periods between waking and sampling, over the weeks. As a result, and because milk cortisol concentrations are generally higher early in the morning, milk cortisol concentrations in women having high maternal psychosocial distress may have been underestimated in this study.”*

*We additionally added the following recommendation for future studies on line 597: “When working with women with clinical diagnoses, extra attention should be given to instructions and reminders, in order to standardize sample collection times.”*
